# Redox Status, JA and ET Signaling Pathway Regulating Responses to *Botrytis cinerea* Infection Between the Resistant Cucumber Genotype and Its Susceptible Mutant

**DOI:** 10.3389/fpls.2020.559070

**Published:** 2020-09-25

**Authors:** Yuting Yang, Xuewei Wang, Panpan Chen, Keke Zhou, Wanyu Xue, Kan Abid, Shuxia Chen

**Affiliations:** ^1^ College of Horticulture, Northwest A&F University, Shaanxi Engineering Research Center for Vegetables, Yangling, China; ^2^ Department of Horticulture, The University of Haripur, Haripur, Pakistan

**Keywords:** cucumber, *B. cinerea*, resistant, redox status, signaling pathway

## Abstract

*Botrytis cinerea* is an important necrotrophic fungal pathogen with a broad host range and the ability to causing great economic losses in cucumber. However, the resistance mechanism against this pathogen in cucumber was not well understood. In this study, the microscopic observation of the spore growth, redox status measurements and transcriptome analysis were carried out after *Botrytis cinerea* infection in the resistant genotype No.26 and its susceptible mutant 26M. Results revealed shorter hypha, lower rate of spore germination, less acceleration of H_2_O_2_, O_2_^-^, and lower total glutathione content (GSH+GSSG) in No.26 than that in 26M, which were identified by the staining result of DAB and NBT. Transcriptome data showed that after pathogen infection, a total of 3901 and 789 different expression genes (DEGs) were identified in No.26 and 26M respectively. These DEGs were highly enriched in redox regulation pathway, hormone signaling pathway and plant-pathogen interaction pathway. The glutathione S-transferase genes, putative peroxidase gene, and NADPH oxidase were up-regulated in No.26 whereas these genes changed little in 26M after *Botrytis* *cinerea* infection. Jasmonic acid and ethylene biosynthesis and signaling pathways were distinctively activated in No.26 comparing with 26M upon infection. Much more plant defense related genes including mitogen-activated protein kinases, calmodulin, calmodulin-like protein, calcium-dependent protein kinase, and WRKY transcription factor were induced in No.26 than 26M after pathogen infection. Finally, a model was established which elucidated the resistance difference between resistant cucumber genotype and susceptible mutant after *B. cinerea* infection.

## Introduction

Gray mold caused by *B. cinerea*, which is a plant fungal pathogen with a typically necrotrophic lifestyle. *B. cinerea* could attack more than 200 plant species including tomato, potato, oilseed rape, kiwi fruit, cucumber, and other high-value as well as important economic crops (Thomma, 2003; Williamson et al., 2007). Cucumber (*Cucumis sativus* L.) is a warm season vegetable crop with suitable growing temperature between 15–32°C and it is mainly cultivated in greenhouse in China (Li et al., 2019). To increase the production and economic efficiency, most of the farmers grow their vegetables continuously in greenhouse that may cause continuous cropping obstacle and ultimately the accumulation of pathogens such as *B. cinerea* (Li et al., 2016). High humidity with 20–30°C temperature inside the greenhouse is suitable for the epidemic *B. cinerea* (Kozhar and Peever, 2018). This fungus can kill the host cells through the production of toxins and acquire nutrients from death cells, thus, a parasitic relationship starts with infection process.

Necrotrophic pathogen infection triggers plant immunity with further biological and physiological processes (Mengiste, 2012). Normally, necrotrophic pathogens are shown to be facilitated by the onset of the hypersensitivity (HR), such as host cell death (van Kan, 2006; Williams et al., 2011), production of various secondary metabolites (Sanchez-Vallet et al., 2010), hormones changes (Garcia-Andrade et al., 2011; La Camera et al., 2011; Duan et al., 2014) and the accumulation of reactive oxygen species (ROS) (Lehmann et al., 2014; Survila et al., 2016). Previously, it has been reported that ROS play a major role in plant and pathogen interactions (Wang et al., 2019), and the hydrogen peroxide (H_2_O_2_) content of the plants increased by regulating the redox signaling pathways after pathogens infection, (Foyer and Noctor, 2005). The level of cellular ROS was one of the cellular redox status markers, and the higher the ROS level, the more oxidizing the cellular redox environment was (Martinez-Sanchez and Giuliani, 2007). Many signaling molecules are important in plants defense response and redox sensitiveness. However, ROS caused severe damage even plant cell death when it accumulated to toxic levels (Baxter et al., 2014). In order to adjust the cellular redox status of some molecules, plants initiated their corresponding scavenging systems (enzymatic and non-enzymatic) to scavenge high intracellular ROS levels which damage the plant integrity (Finiti et al., 2014). Non-enzymatic system has been reported an important one which mainly depended on glutathione (GSH) and ascorbic acid to maintain the intracellular redox homeostasis *via* AsA-GSH cycle (Smirnoff, 2007; Foyer and Noctor, 2011). AsA remove the H_2_O_2_ and eliminate oxidation damage in the cell. First, AsA was oxidized into dehydroascorbate (DHA) under the action of ascorbate peroxidase (APX; EC 1.11.1.11), and then the DHA reduced to dehydroascorbate reductase (DHAR; EC 1.8.5.1). During redox regulation, the glutathione disulfide (GSSG) was converted into GSH which could be recycled through the action of glutathione reductase (GR) using reduced NADPH as the electron donor (Apel and Hirt, 2004).

As the most commonly known members of phytohormones, Jasmonic acid (JA) signaling pathways were reported to have an active effect on inhibiting gray mold (Liu et al., 2019). It was generally believed that activating of JA signaling pathway can induce resistance against necrotrophs (Liu et al., 2019) and endogenous contents of JAs was rapidly up-regulated and then triggered the formation of COI1-JA-JAZ ternary complex to induce degradation of the Jasmonate-ZIM domain (JAZ) proteins (Zhang et al., 2019). The basic helix-loop-helix (bHLH) transcription factor MYC2 acted as a key regulator in the JA signaling pathway (Boter et al., 2004; Lorenzo et al., 2004; Dombrecht et al., 2007) and transcriptional activity of MYC2 was repressed by JAZ (Chini et al., 2007; Thines et al., 2007; Pauwels et al., 2010).

Ethylene (ET) has been reported to play crucial roles in both basal and race-specific immunity (Liu et al., 2018) and found that it plays a crucial role in defense response against *Botrytis* (Audenaert et al., 2002; Belkhadir et al., 2012), especially the ethylene biosynthesis and signaling transduction was induced in response to *B. cinerea* (Nambeesan et al., 2012). Indeed, the activation of ethylene biosynthesis and production under pathogen infection was an early resistance response of the plants (Chagué et al., 2006). In plants, ethylene was synthesized from the amino acid ‘methionine’ through the intermediate aminocyclopropane-1-carboxylic acid (ACC) pathway (Chagué et al., 2006), and it was reported that ACC synthase was the rate-limiting enzyme of ethylene biosynthesis in plants (Liu and Zhang, 2004). ET signals transduction components such as ethylene response factors (ERFs) and ethylene insensitive (EIN) were often involved in plant resistance to *B. cinerea* (Wang et al., 2015).

The induction of phenylpropanoid biosynthesis is a well-known response of plants to of *B. cinerea* infection (Dixon et al., 2002; Wei et al., 2017; Oliva et al., 2020), which provided physical and chemical barriers such as reinforcement of plant cell walls (Barber and Mitchell, 1997). A variety of phenylpropanoid-based polymers including coumarate, ferulate, kaempferol, phenylethyl iso-thiocyanate, 3-phenyllactate, and salicylate were significantly synthesized under the infection of *B. cinerea via* the catalyzing of phenylalanine ammonia-lyase (PAL) and cinnamoyl-CoA reductase (CCR) (Naikoo et al., 2019). The related genes encoded the enzymes involved in the phenylpropanoid biosynthetic pathways were induced (Lloyd et al., 2011). The accumulation of phenylalanine derivatives reduced susceptibility of *Arabidopsis* as well as petunia to *B. cinerea* (Oliva et al., 2020). Lignin biosynthesis is a downstream branch of the phenylpropanoid pathway and the increased expression of genes in lignin biosynthesis has been noted recently, which can thick cell walls to form a physical barrier and inhibit pathogen invasion and colonization (Lee et al., 2019). At the same time, accumulation of lignin also inhibits the spread of pathogens and prevents the pathogens from extracting nutrients from host plants (Mottiar et al., 2016). Thereby, phenylpropanoid-derived metabolites are important in defense against *B. cinerea*.

Cucumber had a narrow genetic background with multiple uses in fresh and processed products, so it was critical important to develop some specialized genotypes for research of genetic modification and innovation, such as disease resistance, stress tolerance, nutritional quality and plant architecture. The available variations for cucumber could be induced by EMS at present. In our study, the resistant genotype and its susceptible EMS-induced mutant were used to investigate the mechanism that regulated responses to *B. cinerea* infection using histological and transcriptome analysis. Differential gene expressions associated with redox regulation and hormone signaling transduction network governing the cucumber defense response and its discovery mechanisms to *B. cinerea* infection were dissected. It will be helpful to uncover the redox status, JA/ET signaling pathway and plant-pathogen interaction pathway regulated in response to *B. cinerea* infection between the resistant genotype and its susceptible mutant. Our study will also lay a foundation for the mechanism more clearly in regulating resistance to *B. cinerea* and will provide a strategy to explore plant defense responses to *B. cinerea* on other horticultural crops.

## Materials and Methods

### Plant Material and Growth Conditions

The plant materials used in this study were provided by our Cucumber Research Group, College of Horticulture, Northwest A&F University, P.R. China and they were the important parental lines in our research group. Cucumber resistant inbred line No.26 and its susceptible mutation 26M induced by EMS were used. The mutant used in our study is the fifth generation of self-breeding seedlings. No significant difference in the growth rate of these two cucumber lines was found, even at seedling stage or maturity stage during the growth process. Seedlings were grown in a controlled growth chamber with a temperature maintained 24 ± 2°C, 16 h light/8 h dark photoperiod and 80%–90% relative humidity. Seedlings having two true leaves were used for pathogen inoculation.

### Fungal Pathogen and Preparation of Spore Suspension

*B. cinerea* was isolated from cucumber plants in greenhouse of Yangling, Shaanxi, China. The pathogens were identified by analyzing their spore morphology and gene sequence. The primer of internal transcribed spacer (ITS1: CTTGGTCATTTAGAGGAAGTAA, ITS4: TCCTCCGCTTATTGATTATGC) was used to molecular identification. Reinfection experiment was carried out in the normal cucumber plant. For conidial production, *B. cinerea* was cultured on potato glucose agar medium at 22°C for 15–20 days in darkness. After 2 weeks, the spores were harvested and filtered through sterile medical gauze. Spores were counted with a haemocytometer and adjusted to 1×10^4^ conidia ml^-1^ using distilled water and 2% sucrose was added as a source of nutrient to start the infection (La Camera et al., 2011).

### Inoculation of Seedlings

Seedling were sprayed with spore suspension until leaf dripping using sprayer, while control plants were sprayed with same amount of sterile distilled water. After inoculation, seedlings were kept in a growth cabinet having same growing conditions as mentioned earlier for 2 days. Samples were harvested at 0, 12, 24, 48 hpi. The experiment was replicated three times and at least five seedlings per replicate were used. The collected samples were immediately frozen in liquid nitrogen and stored at -80°C for further analysis.

In order to assess for disease resistance of seedlings, the 20 μl spore suspension containing 10^4^ spores ml^-1^ were dropped on two sides of the mid-vein of the leaf (La Camera et al., 2011). Equal volume ddH_2_O were dropped as control. Lesion size was measured at 3, 4, 5, and 6 days post-inoculation (dpi). Leaves were harvested at 4, 12, 16, 20, 24, 48, 72, 120 hpi for staining.

### Staining Methods

H_2_O_2_ was stained using 3, 3-diaminobenzidine-tetrahydrochloride (DAB) (Sigma, D8001) according to Orozco-Cardenas and Ryan (1999) in order to observe conveniently. O_2_^−^ was stained using nitro blue tetrazolium (NBT) (Sigma, N6639) according to Carol et al. (2005). The KOH-aniline blue fluorescence staining was used to observe the development of the fungal spores according to Hood and Shew (1996). The fungi were observed using Olympus BX-51 inverted fluorescence microscope (Olympus, Japan) under ultravioletray light.

The spore germination rate and length of the hyphae were observed at 48 hpi. Image was analyzed using Image J.

### Measurement of Glutathione Content

Determination of total glutathione was carried out according to Rahman et al. (2006) with a little bit modifications. Briefly, 0.1 g cucumber leaf was weighted and ground in liquid nitrogen. The reaction volumes were containing 20 μl of KPE buffer (0.1M potassium phosphate buffer with 5mM EDTA disodium salt, pH 7.5), 20 μl clear supernatant, 120 μl equal volume mixture of freshly prepared 0.06% DTNB [5,50-dithiobis (2-nitrobenzoic acid); Sigma] in KPE and 3 U of GR (Sigma) in KPE, and 60 μl NADPH (Sigma). The solution of the reactions displayed yellow. The absorbance was immediately read at 412 nm in a microplate reader.

For GSSG assay, 4 μl of 2-vinylpyridine (Sigma) was added to 20 μl of clear supernatant and mixed well to produce GSH. The reaction was allowed to proceed for 1 h at room temperature in laboratory fume hood. The derivative samples were assayed by the same method as described for total GSH. The total GSH and GSSG concentrations in samples were calculated using a standard curve of GSH and GSSG (containing 2 ml of 2-vinylpyridine) (Sigma).

### Determination of H_2_O_2_ and O_2_^-^

Determination of the H_2_O_2_ referred to the method of Khan et al. (2014). Absorbance was measured at 390 nm by full-wavelength microplate reader (Infinite M200 Pro, Switzerland) and 3 repetitions per treatment. A standard curve was prepared using the H_2_O_2_ standard solution, and the H_2_O_2_ content of the sample was calculated from the standard curve.

The determination of the O_2_^-^ content was carried out by the method of Liu and Wang (2012) with some modifications. Absorbance was measured at 530 nm by full-wavelength micro plate reader (Infinite M200 Pro, Switzerland) and 3 repetitions per treatment. At the same time, a standard curve was prepared using the NaNO_2_ standard solution to calculate the concentration of O_2_^-^.

### Transcriptome Analysis and Functional Annotation

Leaf samples from the control and treated plants were collected at 48 hpi, frozen immediately in liquid nitrogen and stored at -80°C. Three seedlings was used as one replicates and three replicates are included. Samples were sent to Novogene in Beijing (https://en.novogene.com) for RNA sequencing. The reference genome for transcriptome analysis was obtained from http://cucurbitgenomics.org/organism/2.

For deferentially expressed genes (DEGs) analysis, the original read count was normalized. Then the statistical model was used to calculate the hypothesis test probability (*p* value). Multiple hypothesis test corrections were performed to obtain False Discovery Rate (FDR) values. The statistically significant DEGs (P-value < 0.05; |log_2_ FC| > 0) of the resistant versus susceptible cucumber comparison were used to identify enriched Gene ontology (GO) terms. Cucumber gene locus IDs identified through the DEG analysis was used to perform gene ontology (GO) enrichment analysis. RNA-seq raw data are available under the website of NCBI ((https://www.ncbi.nlm.nih.gov/) with the BioProject ID PRJNA630950.

### Validations of DEGs Using Quantitative Real-Time PCR

To confirm the accuracy and reproducibility of the Illumina RNA-Seq results, six candidate DEGs were selected to be tested using qRT-PCR. The leaf of the resistant No.26 and susceptible 26M plants were sampled at 48 hpi, frozen immediately in liquid nitrogen and stored at -80°C. Total RNA was extracted using EasyPure™ Plant RNA kit (TransGen Biotech, Beijing, China). Genomic DNA was digested by using *DNase* I (Thermo Fisher Scientific, USA). cDNA was synthesized using cDNA synthesis kit (TaKaRa Biotechnology, Dalian, China) and quantified with a spectrophotometer (NanoDrop Technologies, Inc.)

All qRT-PCR was run with SYBR green (TaKaRa Biotechnology, Dalian, and China) with a QuantStudio^®^5 real time-PCR machine (Life Technologies, USA). 10 μl of Mix, 0.6 μl of both the forward and reverse gene specific primers (**Table 1**), 2 μl of cDNA, and 6.8 μl of RNase-free water were included per reaction. The process for PCR amplification was as follows: initial denaturation at 95°C for 10 min, followed by 40 cycles at 95°C for 30 s, 56°C for 30 s, and 72°C for 15 s. *CsUBQ5* was used as reference gene to normalize transcript level for each sample, and the final data was calculated using formula 2^−ΔΔCT^.

**Table 1:** The primers of gene for qRT-PCR.

Primer	Sequence
*CsUBQ5*-RT-F	TGGACTCTGGTGATGGTGTTA
*CsUBQ5*-RT-R	CAATGAGGGATGGCTGGAAAA
*Csa3G135120*-RT-F	CTGCATTCATCTCCCCGCTT
*Csa3G135120*-RT-R	AGGCCGTAAAGAAGCATTTCCT
*Csa3G389850*-RT-F	CGATTCAGATTTCTCGACGGAT
*Csa3G389850-*RT-R	AGTTTTCTAGCGCACTGTGGAT
*Csa4G286960*-RT-F	ATGGCGTCTAACATCATTGCAATTG
*Csa4G286960*-RT-R	TTGCTGCCCATCTCTGTCCAAATCT
*Csa6G160180*-RT-F	CAATCATCAACTTGGAGAAGCTTAAT
*Csa6G160180*-RT-R	CTATATCTATTCCGTGGTTCAGTAC
*Csa4G285730*-RT-F	TGGCGGCTTCTTCTAAAGTTATTGT
*Csa4G285730*-RT-R	TGCACAATGCTTGATACGTTAGGAC
*Csa4G045070*-RT-F	CTACGTTTGTGGAGCCACCGAGC
*Csa4G045070*-RT-R	TCGGTTAATCGAAGAACACACTACA

### Statistic Analysis

All data were analyzed using SPSS 20 (Statistical Package for the Social Sciences, Chicago, IL, USA) for Windows PC. Values were presented as mean ± standard deviations (SD). Statistical significance was calculated using Student’s t-test and one-way ANOVA with Duncan’s multiple range test (P<0.05). The statistically significant difference (p<0.05) were presented using asterisk between different genotypes. Histograms were generated using Graph Pad Prism5 (Graph Pad Software Inc., San Diego, CA, USA, 2005). The Heatmap were generated with Heml (version 1.0.3.7) and MeV (Multi Experiment Viewer 4.7.4, TIGR, China) to show the expression patterns of genes.

## Results

### The Lesion Development and Growth of *B. cinerea* Between the Susceptible and Resistant Cucumber Genotypes

**Figure 1:**
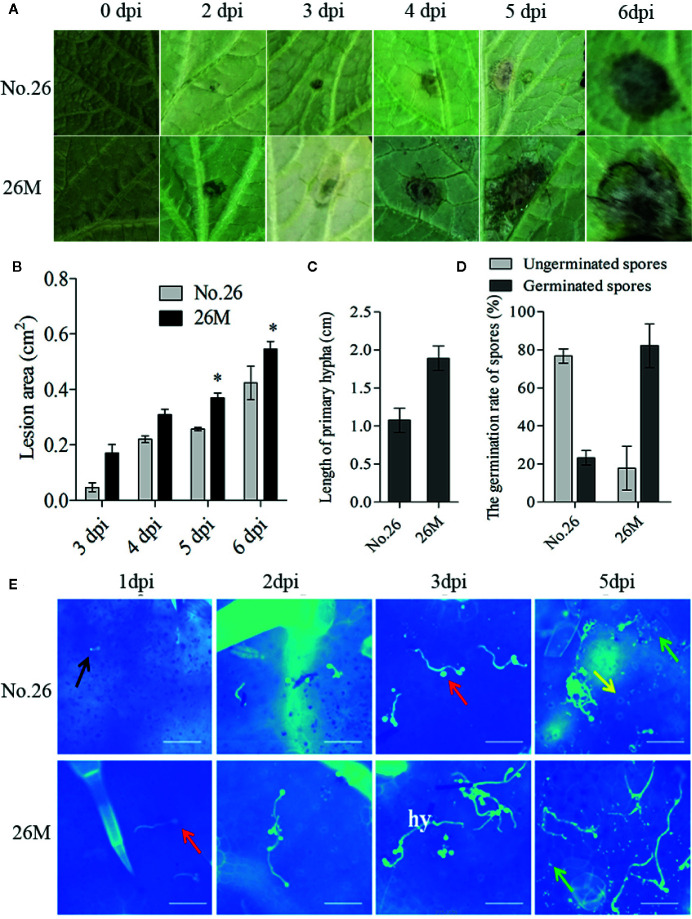
Symptom development in susceptible (26M) and resistant (No. 26) cucumber. **(A)** Disease symptoms observed at 0, 2, 3, 4, 5, 6 days post-inoculation (dpi). **(B)** The lesion area at 3, 4, 5, 6 days post-inoculation (dpi) in susceptible and resistant cucumber; **(C)** The length of primary hyphal at 2 days post-inoculation (dpi) in susceptible and resistant cucumber; **(D)** The germination of spores at 1 day post-inoculation (dpi) in susceptible and resistant cucumber. **(E)** Growth of *B. cinerea* was observed at 1, 2, 3, 5 days post-inoculation (dpi). Red arrow: conidium; Yellow arrow: stomata; Black arrow: germ tube; Green arrow: callose; hy: hypha; Fifteen plants per cucumber genotype were infected with *B. cinerea* and lesion sizes were measured. The means of lesion size data from five independent experiments are shown (mean ± SD). Five seedlings per replicate and three replicates are included for staining. Asterisk indicated the statistically significant difference between different cucumber genotypes after *B. cinerea* infection (p < 0.05). Bar=50 μm.

Lesion development was observed after infection by the identified *B. cinerea* (**Figure S1**). Visible lesions were first observed on the back side of leaves in both cucumber lines at 48 hpi. Then the lesions on the leaves of the two cucumber genotypes became water-soaked and macerated, displayed a typical spreading symptom (**Figure 1A**). The lesion area was measured. The lesion area ranged from 0.047 to 0.42 cm^2^ in No.26 whereas the lesion area changed from 0.17 to 0.55 cm^2^ in 26M (**Figure 1B**). A significant difference in the lesion area of both genotypes was observed at 5 days and 6 dpi.

In order to observe the germination and growth of *B. cinerea* on the inoculated leaves, 10 μl spore suspension were dropped both sides of the leaf beside the main vein. The conidia of *B. cinerea* germinated at 24 h post-inoculation (hpi) and the developed mycelia get elongated after 48 h post-inoculation (hpi) (**Figure 1E**). After penetration of the cuticle the fungus hypha formed and elongated at 48 hpi. Hyphae continuously elongated with invasion progresses (**Figure 1E**). We also measured the rate of spore germination and length of primary hypha at 48 hpi (**Figures 1C, D**). The result showed that the wild type No.26 had lower spore germination rate and shorter mycelia than that in 26M. The rate of spore germination was individually 23.23% in No.26 and 82.16% in 26M. The mean length of primary hypha was about 1.07 cm in No.26, while the mean length of primary hypha was about 1.89 cm in 26M.

### The Difference in the Content of H_2_O_2_ and O_2_^-^ Between Infected Leaves of No.26 and 26M

**Figure 2:**
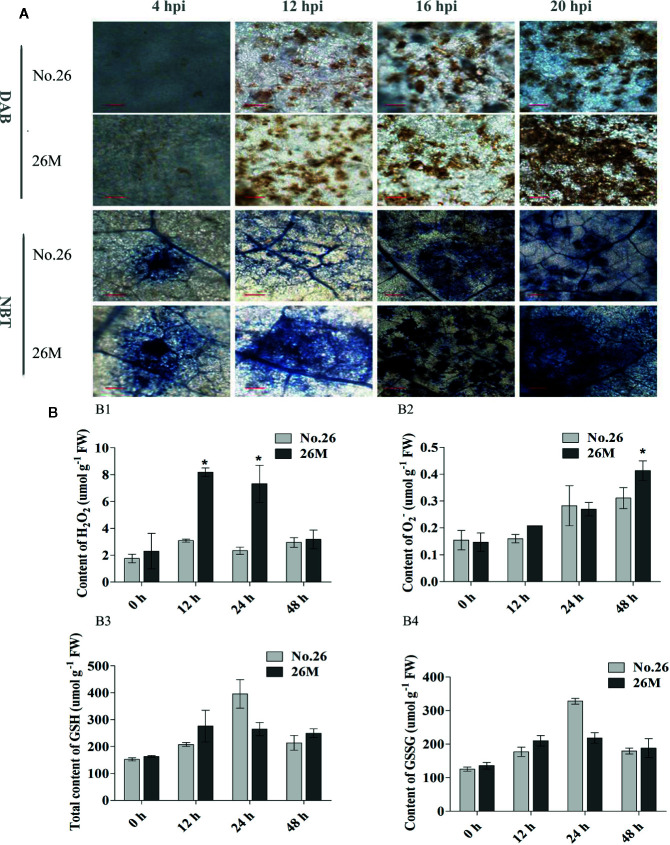
Content of hydrogen peroxide, superoxide anion. **(A)** DAB and NBT staining; **(B1)** the content of H_2_O_2_; **(B2)** the content of O_2_^-^; **(B3)** the content of total glutathione and **(B4)** the content of oxidized glutathione. At least five seedlings per replicate and three replicates are included for determining content of H_2_O_2_ and O_2_^-^. Asterisk indicated the statistically significant difference between different cucumber genotypes after *B. cinerea* infection (p < 0.05). Bar=50 μm (at 4 hpi). Bar=200 μm (at 12 hpi, 16 hpi, 20 hpi).

Quantitative measurements of ROS were carried out and the microscopy detections of superoxide ion (O_2_^-^) and hydrogen peroxide (H_2_O_2_) were observed using nitro blue tetrazolium (NBT) and 3, 3’-diaminobenzidine (DAB) staining respectively. It showed that the enhanced synthesis of O_2_^-^ and H_2_O_2_ were observed in the leaves after inoculation of *B. cinerea*. The H_2_O_2_ accumulation was observed in the early time of inoculated leaves of both 26M and No.26 at 4 hpi (**Figure 2A**), and the significant difference between them was observed at 12 hpi. Accumulation of H_2_O_2_ in the leaves of 26M was significantly higher than that in the leaves of No.26 at 12 dpi and 24 hpi (**Figure 2B1**).

The production of O_2_^-^ was higher at 4 hpi and 12 hpi in the inoculated leaves of 26M than No.26 (**Figure 2A**). During the infection, an obvious difference in the production of O_2_^-^ was observed between the 26M and No.26 genotypes (**Figures 2A, B2**). The O_2_^-^ concentration in the inoculated leaves of both genotypes reached the maximum at 48 hpi, and was significantly higher in 26M than No.26 (**Figure 2B2**).

### The Difference of GSH Content Between Infected Leaves of No.26 and 26M

GSH participated in the regeneration of ascorbate *via* dehydroascorbate reductase (DHAR), thus contributing to H_2_O_2_ detoxification (Smirnoff, 2007). The total GSH content increased after the seedlings were inoculated with suspension of pathogen (**Figure 2B3**) and peaked at 24 hpi in both 26M and No.26 genotypes. The content of total GSH in 26M was much higher than that in No.26 at 12 hpi and 48 hpi after *B. cinerea* infection. The content of total GSH in 26M was 1.33 and 1.17 folds respectively as compared with No.26. The content of GSSG showed the similar trend with total GSH content which was significantly increased with the infection time in both No.26 and 26M genotypes and peaked at 24 hpi (**Figure 2B4**). This suggests an effect of fungi treatment on the oxidative imbalance.

### Differential Expression Genes Between 26M and No.26 After *B. cinerea* Infection

**Figure 3:**
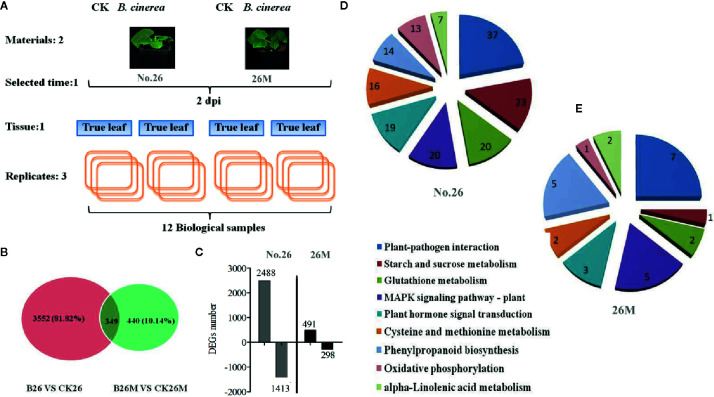
Expression patterns of unigenes differentially expressed after leaf inoculation with *B. cinerea*. **(A)** Experimental design; **(B)** The number of differentially expressed genes after inoculation with *B. cinerea* at 2 days post-inoculation (dpi) in the susceptible and resistant cucumber; **(C)** The number of up and down regulated expression genes after inoculation with *B. cinerea* at 2 days post-inoculation (dpi) in the susceptible and resistant cucumber. **(D, E)** The analysis of metabolite pathway in resistant and susceptible cucumber under *B. cinerea* infection.

A total of 3901 DEGs were identified between No.26 and its control, while 789 DEGs were identified between 26M and its control respectively (**Figures 3A, B**). Among DEGs in 26M, 491 DEGs were significantly up-regulated and 298 DEGs were significantly down-regulated. In resistant No.26 genotype, 2,488 DEGs were significantly up-regulated and 1,413 DEGs were down-regulated in a significant manner (**Figure 3C**). After *B. cinerea* infection, it was noted that there were more up-regulated DEGs than down-regulated in both genotypes and more DEGs were identified in No.26 genotype as compared to 26M genotype. Multiple genes were found to be involved in the interaction between cucumber and *Botrytis* pathogen.

### The Result of KEGG Analysis

Based on the KEGG descriptions of the entire set of DEGs, the number of genes with particular annotation term was significantly overrepresented (**Figures 3D, E**). There were 104 biochemical pathways identified in No.26 compared as compared to the control, in which 37 DEGs were mainly enriched in plant defense responses during plant-pathogen interaction, while 19 important DEGs were identified in hormone signal transduction such as SA, JA and ET. Some other DEGs were found to be enriched in the MAPK signaling pathway, the redox regulation and phenylpropanoid biosynthesis in No.26 compared with that in control (**Figure 3D**). Whereas there were fewer DEGs identified in susceptible 26M, in which only seven DEGs were involved in plant-pathogen interaction, five and three DEGs involved in MAPK signaling and phytohormones signal transduction pathway respectively, two DEGs were found to be involved in glutathione metabolism and 5 were involved in phenylpropanoid biosynthesis (**Figure 3E**).

### Differential Expression Genes Related to Redox Status

**Figure 4:**
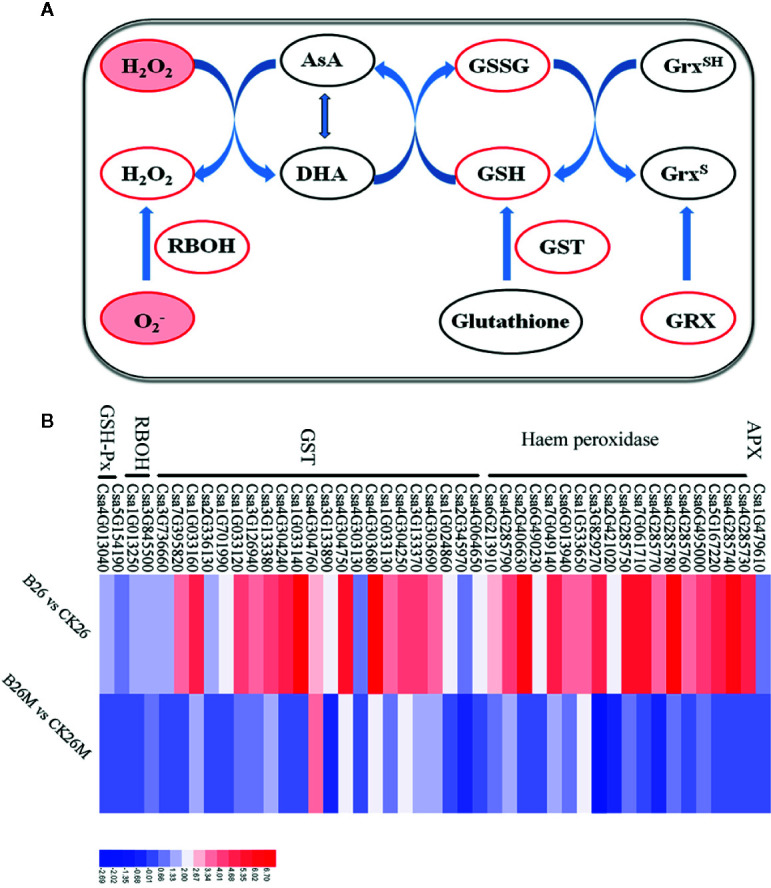
Differentially expressed genes related to ROS regulation at 2 days post-inoculation (dpi) in the susceptible and resistant cucumber **(B)** and some key substances involved in ROS regulation **(A)**. RBOH: respiratory burst oxidase homologue; GST: glutathione S-transferase; GRX: glutaredoxin; AsA: ascorbate and DHA: dehydroascorbate. Red box represented up-regulation of genes or increment in content.

The oxidative burst caused by the hypersensitive response following perception of pathogen virulence signals is an early plant response to pathogen infection, which often led to rapid generation of superoxide, accumulation of H_2_O_2_ and the hypersensitive response (Torres et al., 2006; Patykowski, 2006; Asai and Yoshioka, 2009; Foyer and Noctor, 2013). After pathogen infection, some DEGs related to redox status regulation were identified, which were mainly involved in the up-regulation of some key antioxidant enzymes, such as peroxidases, glutathione S-transferase (GSTs), acerbates oxidase, superoxide, and NADPH oxidase in both 26M and No.26 genotypes (**Figure 4**). Four glutathione S-transferase genes (*Csa4G303690*, *Csa3G126940*, *Csa4G304250*, and *Csa4G304760*), one putative peroxidase gene (*Csa7G049140*) and a NADPH oxidase gene (*Csa3G845500*) were significantly up-regulated in resistant No.26 genotype compared with susceptible 26M genotype, indicating that resistant genotype No.26 prevented oxidative damage caused by *B. cinerea*. Several glutathione S-transferase genes such as *Csa1G033160*, *Csa7G395820*, *Csa1G701990*, *Csa1G033130*, *Csa1G033140*, and peroxidase genes including *Csa4G304750*, *Csa4G285740*, *Csa6G013940*, *Csa4G285760*, *Csa4G285770* were up-regulated in resistant No.26 genotype (**Figure 4B**), whereas these genes changed little in susceptible 26M mutant. Other oxidoreductases were also identified such as glutaredoxin (*Csa6G518190*) which is involved in redox regulation, was induced in susceptible genotype 26M, whereas no change was found in No.26 genotype. Glutaredoxins (GRXs) played a crucial role in response to oxidative stress by reducing disulfides in various organisms, and it has been reported that *A. thaliana* glutaredoxin *ATGRXS13* was required to facilitate *B. cinerea* infection (La Camera et al., 2011). All of these results suggested that rapid redox regulation in the resistant genotype responding to the infection of pathogen was necessary for the resistance.

### Differential Expression Genes Related to JA

**Figure 5:**
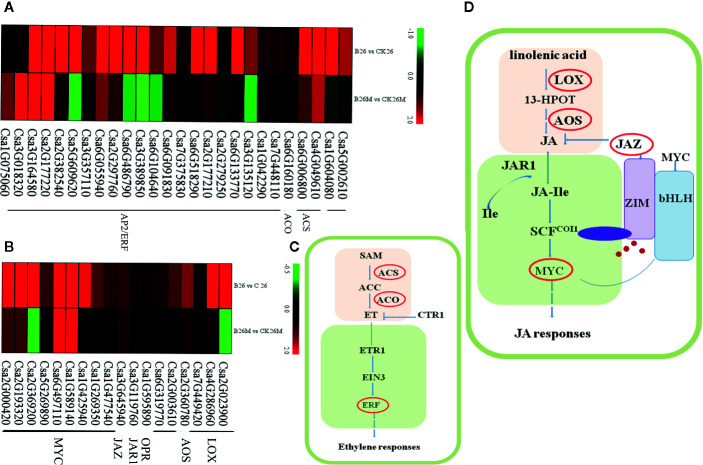
Differentially expressed genes involved in hormone signal pathway at 2 days post-inoculation (dpi) in the susceptible and resistant cucumber. DEGs in ET signal **(A)** and JA signal **(B)**. **(C)** Simplified ET signal transduction induced by *B. cinerea* infection. **(D)** Simplified JA signal transduction induced by *B. cinerea* infection. 13-HPOT (13(S)-9(Z), ll(E), 15(Z)-Hydroperoxyoctade-catrienoic acid); LOX (Lipoxygenase); JA (jasmonate); JAR1 (jasmonate resistant 1); SCF (Skp/Cullin/F-box); COI1 (coronatine-insensitive 1); JA-Ile (jasmonate-isoleucine); JAZ (jasmonate ZIM domain protein); MYC (MYC transcription factor); SAM (S′ adenosyl-l-methionine); ACS (1-aminocyclopropane-1-carboxylic acid synthase); ACC (1-aminocyclopropane-1-carboxylic acid); ACO (1- aminocyclopropane-1-carboxylate oxidase); ET (ethylene); ETR1 (ethylene-response gene 1); CTR1 (constitutive triple response); EIN3 (ethylene insensitive 3); ERF (ethylene-responsive transcription factor). Red box represented up-regulation of genes.

Jasmonate acid (JA) plays an important role in the defense response of plants against fungal pathogens. Genes encoding of Lipoxygenase (LOX), allene oxide synthase (AOS), and alcohol dehydrogenase (ADH) involved in JA synthesis pathway were identified and the jasmonate resistant 1 (JAR1), jasmonate ZIM domain protein (JAZ) and MYC transcription factor (MYC) genes involved in JA signaling pathway were identified after pathogen infection (**Figures 5B, D**). Some genes which were closely related to JA synthetic pathway were significantly up-regulated in No.26, such as one ADH (*Csa7G322060*), three oxidase reductases (*Csa1G595890*, *Csa6G319770*, and *Csa2G003610*), while only *LOX* (*Csa7G449420*) and *AOS* (*Csa2G360780*) were up-regulated in 26M, indicating that the synthesis of JA was inhibited in some extent in susceptible 26M. Eight MYC2 transcription factors (*Csa2G000420*, *Csa1G425940*, *Csa1G589140*, *Csa1G269350*, *Csa5G269890*, *Csa2G193320*, *Csa2G369200*, and *Csa1G477540*) which are the key components of JA signaling pathway were significantly up-regulated in No.26 genotype, while in 26M genotype they were either induced a little or not induced at all, showing that JA signal transduction pathway was inactive in 26M during *B. cinerea* infection.

### Differential Expression Genes Related to ET

Ethylene (ET) is a main modulator of disease resistance in plants. Genes related to both ET biosynthetic and signal transduction pathways were identified during *B. cinerea* infection (**Figures 5A, C**), such as cytosine-specific methyltransferase, 1-aminocyclopropane-1-carboxylic acid synthase (ACS), 1- aminocyclopropane-1-carboxylate oxidase (ACO), ethylene-responsive transcription factor (ERF). After *B. cinerea* infection, the *ACS* (*Csa4G049610*), *ACO* (*Csa6G160180*) and two methyltransferase (*Csa1G604080* and *Csa5G002610*) genes were induced in No.26, whereas *ACO* and methyltransferase genes were not induced in 26M, indicating that synthesis of ET was inhibited in 26M during *B. cinerea* infection. Sixteen ERFs (*Csa6G133770*, *Csa6G091830*, *Csa6G486790*, *Csa2G177210*, *Csa2G297760*, *Csa6G518290*, *Csa3G135120*, *Csa7G375830*, *Csa2G382540*, *Csa7G448110*, *Csa3G389850*, *Csa5G609620*, *Csa1G042290*, *Csa3G357110*, *Csa6G104640*, and *Csa2G279250*) and a MAPK (*Csa3G651720*) gene was significantly up-regulated in resistant No.26 genotype, whereas only five other ERFs involving ET signal transduction were up-regulated in 26M, indicating that the ET signaling transduction pathway in 26M was not as active as that in No.26.

### Differential Expression Genes Related to Phenylpropanoid Pathway

**Figure 6:**
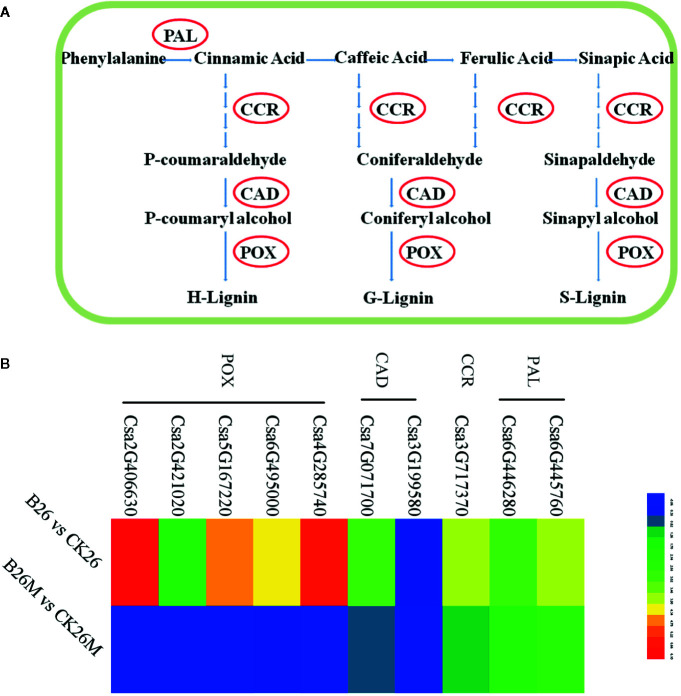
Differentially expressed genes involved in PAL pathway at 2 days post-inoculation (dpi) in the susceptible and resistant cucumber **(B)** and simplified PAL pathway induced by *B. cinerea* infection **(A)** PAL (phenylalanine ammonia-lyase); CCR (cinnamoyl-CoA reductase); CAD (cinnamoyl alcohol dehydrogenase); POX (peroxidase). Red box represented up-regulation of genes.

It was found that there were some DEGs associated with phenylpropanoid pathway to be identified between the resistant and susceptible cucumber genotypes (**Figures 6A, B**). Some genes such as phenylalanine ammonia-lyase (PAL), lignin biosynthetic enzymes, cinnamoyl-CoA reductase (CCR), cinnamyl alcohol dehydrogenase (CAD), and peroxidase (POX) associated with phenylpropanoid pathway were up-regulated during *B. cinerea* infection. However, some genes were only induced in resistant No.26 genotype, such as *CAD* genes (*Csa7G071700* and *Csa3G199580*) and *peroxidase* genes (*Csa4G285740*, *Csa2G406630*, *Csa5G167220*, *Csa6G495000* and *Csa2G421020*), while the transcript level of these genes did not change in susceptible 26M genotype (**Figure 6B**).

### Differential Expression Genes Related to Plant Defense

**Figure 7:**
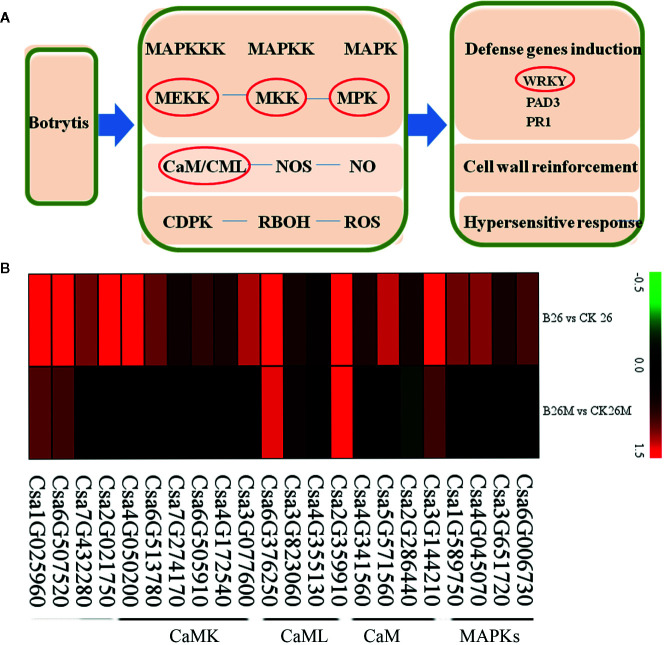
Differentially expressed genes involved in basal defense at 2 days post-inoculation (dpi) in the susceptible and resistant cucumber **(B)** and simplified basal defense induced by *B. cinerea* infection **(A).** MAPK (mitogen-activated protein kinase); CaM (calmodulin), CaML (calmodulin-like protein); CaMK (calcium-dependent protein kinase).

The expression results revealed that some plant defense related DEGs such as mitogen-activated protein kinases (MAPKs), calmodulin (CaM), calmodulin-like protein (CaML), and calcium-dependent protein kinase (CaMK)were identified during *B. cinerea* infection (**Figures 7A, B**). Two MAPK genes (*Csa6G006730* and *Csa4G045070*), two mitogen-activated protein kinase kinase (MAPKK) genes (*Csa1G589750* and *Csa3G651720*) and a mitogen-activated protein kinase kinase (MAPKKK) gene (*Csa2G021750*) were highly up-regulated in No.26, whereas the above mentioned defense-related genes were not induced in 26M. Five CaM*/*CaML genes (*Csa5G571560*, *Csa4G341560*, *Csa4G355130*, *Csa3G144210*, and *Csa2G286440*) and six *CaMK* genes (*Csa6G513780*, *Csa6G505910*, *Csa3G077600*, *Csa2G359910*, *Csa4G050200*, *Csa7G274170*, and *Csa4G172540*) were significantly up-regulated in No.26, while they were not induced in 26M. In addition, glycerol kinase (*Csa7G432280*) and chitinase (*Csa6G507520*) were also up-regulated in No.26. CaM/CaML genes (*Csa3G823060* and *Csa6G376250*) were induced in both genotypes after pathogen infection.

Interesting, the transcription level of 24 WRKY genes had changed in both cucumber genotypes upon *B. cinerea*, (**Figure S2**). Two *WRKY* genes (*Csa2G406060* and *Csa7G049170*) were up-regulated in No.26, whereas these two genes were down-regulated in 26M after pathogen infection. The rest of *WRKY* genes displayed the similar expression pattern in both cucumber lines upon *B. cinerea* infection, which exhibited the higher transcription level in No.26 while lower transcription level in 26M. Among them, three *WRKY* genes (*Csa6G486960*, *Csa5G223070* and *Csa6G363560*) were highly induced in No.26 upon *B. cinerea*, which were more than 6-fold change than control. They were showed lower transcription level in 26M and were about 1-fold change compared with control.

### Validation of Transcriptomic Expression Levels

Six genes were selected to validate the RNA-Seq results through real-time quantitative PCR (qPCR) in leaves of both No.26 and 26M genotypes. These genes included *lipoxygenase* (*Csa4G286960*), AP2 transcription factors (*Csa3G135120* and *Csa3G389850*), *ACO* (*Csa6G160180*), *POD* (*Csa4G285730*), and *MAPK* (*Csa4G045070*) (**Table 1**). The qRT-PCR results were in accordance with the transcriptomic data (**Figure S3**). The transcript level of these genes in resistant cucumber genotype No.26 was higher than susceptible cucumber genotype 26M under *B. cinerea* infection.

## Discussions

### Redox Status Regulation Was Crucial for the Response to *B. cinerea*

There were few reports about the interaction between cucumber and *B. cinerea* by far, so the mechanism about the cucumber resistance to *B. cinerea* was not well elucidated. It was believed that redox-related gene expression and metabolites were activated in the priming of plant and were regulated by proper cellular function as well as enzymatic and non-enzymatic mechanisms (González-Bosch, 2018). ROS production and scavenging networks were composed of ROS-producing enzymes, antioxidant enzymes and biosynthetic pathways of antioxidants (Posmyk et al., 2009), which were crucial in the plant defense against external threats (Noctor et al., 2018). In current study, the regulation of oxidative state caused by *B. cinerea* was significantly different between the resistant and susceptible.

**Figure 8:**
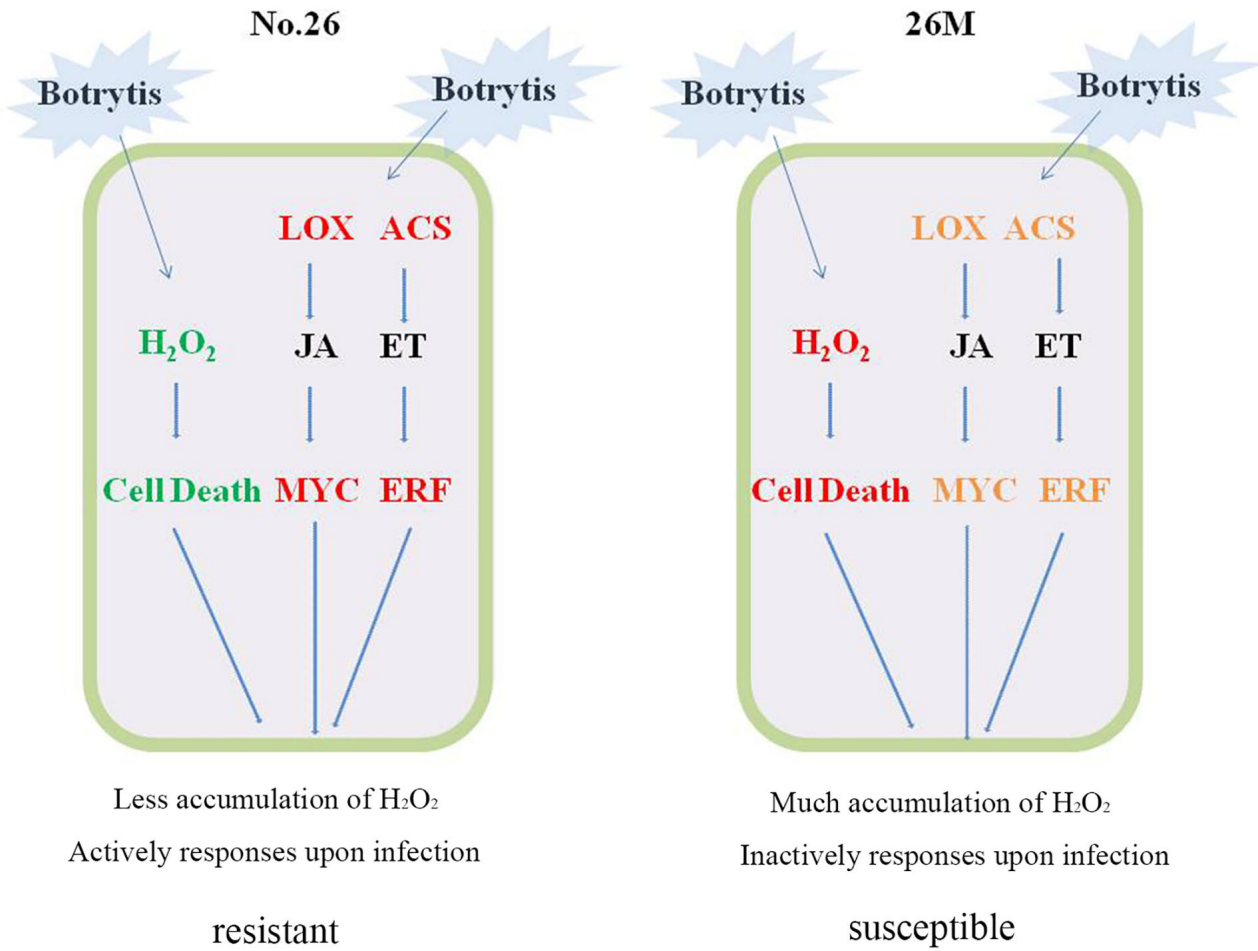
Cellular model summarizing potential mechanism of cucumber resistance to *B. cinerea*. Red represented the higher level of gene expression and much accumulation of content; Green represented the less accumulation of content. Orange represented the lower level of gene expression.

The oxidative burst and scavenging were often regulated by cellular detoxification system such as peroxidases, glutathione S-transferases, ascorbate oxidase, superoxide, and NADPH oxidase (Heller and Tudzynski, 2011). These activated antioxidant and detoxifying enzymes were activated and induced by the infection of pathogens in the invaded plant cells (Lamb and Dixon, 1997; AbuQamar et al., 2006; Parisy et al., 2007). In our study, it was found that some genes encoding antioxidant and detoxifying enzymes such as glutathione S-transferases (GSTs), peroxidase and other antioxidant enzymes were significantly induced in No.26, while these genes were not induced so obviously in the 26M genotype during *B. cinerea* pathogen infection (**Figure 4**). There were some other genes regarding glutathione S-transferases (GSTs) and peroxidase which were induced in the resistant cucumber genotype than in susceptible genotype, signifying that more ROS were removed in No.26 and less ROS were accumulated (**Figure 2**). Normally, ROS such as H_2_O_2_ and O_2_^-^ occurred at early stage during infection of necrotrophics, which caused cell death or as an indicator of successful infection and colonization of necrotrophic pathogens (Govrin and Levine, 2000; Govrin et al., 2006; van Kan, 2006). The amount of ROS affected the extent of programmed cell death and the colonization of pathogens, which in turn affected the resistance of plant to pathogens (**Figure 8**).

GSH, belong to non-enzymatic antioxidant system, that can reduce ROS by directly reaction with it. GSH can also play an important role in the removal of ROS as a substrate for the enzymes. GSH and redox equilibrium-controlling genes were essential for an appropriate defense response against *B. cinerea* (Chassot et al., 2008). High content of total GSH and GSSG were detected after *B. cinerea* infection in both cucumber genotypes used in our study, reflecting a rapid regulation of oxidized status at this time (**Figure 2**). The result was consistent with the early oxidative burst which occurred on fungal inoculation (Finiti et al., 2014).

### Multiple Hormone Signaling Pathways Modulated Resistance Responses to *Botrytis* Pathogens

Plant always defends itself through fine-tuning of the hormones to cope with biotic and abiotic stresses (Miao et al., 2019). JA, SA, and ET were thought to be main phytohormones in response to pathogen attack. It was generally believed that salicylic acid (SA) was traditionally associated with defense against biotrophic and hemibiotrophic pathogens, whereas jasmonic acid (JA) and ethylene (ET) signaling appear to be more important towards necrotrophic pathogens and herbivores (Erb et al., 2012; Mengiste, 2012; Pieterse et al., 2012; Han and Kahmann, 2019). It has been reported that the synthesis of JA was activated by the infection of *B. cinerea* through the upstream synthesis gene *AdLoxD* of tomato. Besides, the ET synthetic pathway was also elicited by the up-regulation of the ET-forming aminocyclopropane-1-carboxylic acid oxidase gene in response to *B. cinerea* (Finiti et al., 2014). There were more and more evidences that the content of JA and ET increased after infection of *B. cinerea*.

The JA/ET signaling pathway was activated against necrotrophs through some key regulators such as transcription factors MYC2 and ERF (Kazan and Manners, 2009; Liu et al., 2018). The *ERF1*, *ERF5*, *ERF6*, *RAP2.2*, and *ORA59* were involved in the regulation of plant defense against *B. cinerea* in *Arabidopsis* (Ouyang et al., 2016; Zhang et al., 2016; Wang et al., 2018; Sham et al., 2019). The resistant ‘Syrah’ grapes displayed higher basal levels of JA-Ile comparing to the susceptible ‘Trincadeira’ along with higher expression of MYC2 and JAZ8 although the synthesis of jasmonic acid increased in infected berries of both ‘Syrah’ and ‘Trincadeira’ cultivars (Coelho et al., 2019). In our study, the synthetic pathway of JA and ET is inhibited to a certain extent in the susceptible material, resulting in partial inhibition of related signal transduction and inactive response to *B. cinerea* infection. This illustrated that the activation of hormone signals of JA/ET and the expression of downstream defense-related genes played an important role against gray mold resistance.

### Resistance Responses to *Botrytis* Infection Was Closely Related With Phenylpropanoid Pathway

Phenylpropanoid pathway was reported to be a pivotal pathway in defending pathogen invasion because of phenylalanine derivatives, which play an active role to defend the pathogen attack (Dixon et al., 2002). PAL, lignin, coumarins, benzoic acids, stilbenes, and flavonoids/isoflavonoids were important derivatives of phenylpropanoid pathway which exhibited broad-spectrum antimicrobial activity and were believed to help the plants to fight against microbial disease (Dixon et al., 2002). The activities of PAL, cinnamate 4-hydroxylase (C4H) and CAD enzyme were differentially induced between the two apple cultivars in response to *B. cinerea* (Ma et al., 2018). In addition, the phenylpropane polymer lignin provided strength to the cell walls for preventing direct penetration of the fungus (Pesquet et al., 2019). CAD as a key enzyme in lignin biosynthesis, the *AtCAD4/5* double mutant *Arabidopsis* exhibited 40% reduction in lignin biosynthesis and was more susceptible to infection (Sibout et al., 2005). Down-regulation of genes encoding PAL, lignin biosynthetic enzymes, ferulate 5-hydroxylase (F5H), cinnamoyl-CoA reductase (CCR) and CAD led to the reduction of resistance to necrotrophic fungi *Sclerotinia sclerotiorum* in resistant soybean line (Ranjan et al., 2019). In our study, the up-regulation of *PAL* gene, lignin biosynthetic enzymes gene, *CCR* gene, *CAD* gene and peroxidase (*POX*) gene were observed to be induced in No.26 resistant genotype, whereas only CCR gene was induced in susceptible genotype 26M, while CAD and POX genes exhibited no change in their expression, indicating that the activation of phenylpropanoid pathway might be the reason of resistance to *Botrytis* infection in the resistant genotype No.26.

### Defense Related Genes Involved in Response to *Botrytis* Infection

Studies showed that MAPKs of tobacco and their orthologs in other species including Arabidopsis MPK3 and MPK6 were activated after PAMP treatment or pathogen infection (Yang et al., 2001; Ichimura et al., 2002), and MAPK cascades were predominately involved in defense when strawberry fruit were infected by *B. cinerea* (Haile et al., 2019). Inhibition of SlMPK1/2/3 disrupted tomato fruit defense signaling pathways and enhances the susceptibility to *B. cinerea*, while knock-down of *SlMAPK3* reduced the disease resistance of tomato to *B. cinerea* (Zhang et al., 2018). Calmodulins (CaM) have been reported to be involved in plant defense responses (Kim et al., 2002; Kang et al., 2006). A Ca^2+^-independent calmodulin-binding proteins (CaMBP) affected defense against the *B. cinerea* in Arabidopsis (Lv et al., 2019). *NtCaM1*, *NtCaM2* and *NtCaM13* were induced by tobacco mosaic virus (TMV)-mediated hypersensitive reaction in tobacco (Takabatake et al., 2007). In our study, five *CaM/CaML* genes and six *CaMK* genes were significantly up-regulated in resistant genotype No.26, while they were not induced in 26M, which confirmed that the activation of *CaM* genes were essential for the resistance response of the No.26 genotype.

Global transcriptional profiling have implicated the involvement of key transcription factors (TFs) in interaction between plant and *B. cinerea (* AbuQamar et al., 2006; Mathys et al., 2012; Windram et al., 2012 *).* These TF families included ERFs (Dey and Corina Vlot, 2015; Zhang et al., 2016), MYBs (Mengiste, 2012), NACs (Nuruzzaman et al., 2013), MYCs (Kazan and Manners, 2013) and WRKYs (Birkenbihl and Somssich, 2011; Jiang and Yu, 2016). These TFs either positively or negatively modulated the plant defenses. Reports showed that the *wrky33* mutant was highly susceptible to *B. cinerea* (Liu et al., 2015). However, loss of function of *WRKY57* has been reported to enhance the host resistance to this pathogen (Jiang and Yu, 2016). Our results also revealed that WRKY transcription factors were involved in *B. cinerea* infection. There were 24 WRKY transcription factors were detected (**Figure S2**). Some of the WRKY transcription factors (*Csa2G406060*, *Csa3G727990*, *Csa6G486960*, *Csa5G223070*, *Csa7G328830*, *Csa4G051470*, and *Csa4G012390*) showed higher transcript level in No.26 cucumber. All of the above-mentioned genes were up-regulated more than 3–6 folds while their expression was only 1–2 fold in 26M compared with uninfected cucumber. *Csa7G049170* and *Csa2G406060* WRKY genes had an opposite expression patterns in both cucumber genotypes, which were induced in No.26 and down-regulated in 26M respectively. These results showed that WRKY transcription factor might be a key factor in resistance to *B. cinerea*, which may regulated the gray mold either negatively or positively in cucumber.

### Genotype-Related Differences in Response to *Botrytis* Infection

Different genotypes exhibited a different response to *B. cinerea*. Transcriptome study of tomato fruits upon *B. cinerea* infection displayed that the genes associated with biosynthesis and signal transduction of ethylene (ET) and jasmonic acid (JA) were involved in interaction between tomato fruit and pathogens (Blanco-Ulate et al., 2013). Same situation was observed on resistant and susceptible grape cultivars. The transcription level of genes involved in the JA signaling pathway was induced after *B. cinerea* infection (Coelho et al., 2019). In our study, similar results were also appeared in both cucumber genotypes upon infection. The infection of *B. cinerea* included accumulation of H_2_O_2_ as well as O_2_^-,^ and up-regulated expression of genes related to synthesis and signal transduction of JA, ET, PAL and defense (**Figures 2**, **5**–**7**).

However, some genotype-related differences were presented. Our results also revealed the effect of genotype on the ROS production and gene expression after *B. cinerea* infection. There were less accumulation of H_2_O_2_ and O_2_^-^ and much more DEGs were induced in cucumber resistant genotype No.26 than that in cucumber susceptible genotype 26M upon infection (**Figures 2B1, B2, 3B**). All of the DEGs were summarized into three categories between No.26 and 26M. The first type was that DEGs had an opposite expression pattern in both cucumber genotypes, which were up-regulated in No.26 and down-regulated in 26M respectively. DEGs were induced in No.26 while their expression was constant in 26M, which was another expression pattern of DEGs. The last type was that expression pattern of DEGs were common in both cucumber genotypes. Difference was mainly focus on the fold change of EDGs. The Higher induced expression fold of DEGs was exhibited in No.26 whereas lower fold change of DEGs was shown in 26M. It indicated that some difference existed between two different cucumber genotypes after infection by *B. cinerea*. There was an exception for the content of total GSH. It was reported that glutathione can act as a powerful cellular redox agent to remove ROS and regulate post-translational modification of protein (Ball et al., 2004; Foyer and Noctor, 2005; Dalle-Donne et al., 2007). Obviously, glutathione initiated oxidative regulation because of increment of glutathione content in both cucumber lines upon *B. cinerea* infection. While measurement of glutathione content was not at later stage of infection but at early stage of infection, which may be the major reason why it was no significant difference in glutathione content between No.26 and 26M after *B. cinerea* infection. Moreover, previous research indicated that GSH also could act as a signaling molecule, which was not only involved in pathogen defense but also actively participate in the cross-communication with other signaling molecules such as JA and ET (Ghanta et al., 2011). Thus, the effect of GSH appeared to be unobvious among the many defense-response signal molecules induced at the early stage of infection in our study. Instead, some WRKY transcription factor might play an important role currently. Especially for the two *WRKY* genes *Csa2G406060* and *Csa7G049170*, they may exert a key function in resistant to *B. cinerea*, because their completely different expression patterns were displayed in No.26 and 26M after *B. cinerea* infection. Other two *WRKY* genes *Csa6G363560* and *Csa6G486960* were also important. As for how these *WRKY* genes affect and regulate plant disease resistance, it needs to be further confirmed by experiment.

## Conclusion

In conclusion, the role of ROS, JA, and ET in response to necrotrophic pathogens was revealed combined ROS content with differential expression genes involved in hormonal synthesis and signal transduction (**Figure 8**). In resistant cucumber genotype No.26, Less acceleration of H_2_O_2_ and O_2_^-^ resulted in the less cell death that was beneficial to facilitate infection of *B. cinerea*. Higher transcription level of differential expression genes having a correlation with JA and ET signaling pathway triggered an active defense response that lead to the tolerance of cucumber No.26 against *B. cinerea.* While cucumber genotype 26M suffered from severe ROS production, accompanied by relative lower or constant transcription level of differential expression genes involved in JA and ET signaling pathway, which result in an inactive response and susceptible to *B. cinerea*.

## Data Availability Statement

The RNA-seq data has been uploaded to NCBI (https://www.ncbi.nlm.nih.gov/). The BioProject ID is PRJNA630950. https://www.ncbi.nlm.nih.gov/bioproject/PRJNA630950/.

## Author Contributions

SC conceived and designed the experiments. YY performed the experiments and contributed to the writing of the manuscript. SC and KA revised the manuscript. YY, XW, and PC analyzed the data. KZ and WX contributed to reagents/materials. All authors contributed to the article and approved the submitted version.

## Funding

This work was supported by the National Natural Science Foundation of China (Grant No. 31772335), Ningxia Major Science and Technology Project during the 13^th^ 5-Year Plan Period (2016BZ0904), and Shaanxi Agricultural Science and Technology Innovation Project (NYKJ-2019-YL04).

## Conflict of Interest

The authors declare that the research was conducted in the absence of any commercial or financial relationships that could be construed as a potential conflict of interest.

## Supplementary Material

Supplementary Figure 1 Isolation and identification of B. cinerea. **(A)** the isolation of B. cinerea; **(B)** morphological observation of B.cinerea; **(C)** PCR amplification; **(D)** sequence alignment; **(E)** symptom of B. cinerea after reinfection in cucumber.

Supplementary Figure 2 The DEGs of WRKY in susceptible (26M) and resistant (No. 26) cucumber at 2 dpi.

Supplementary Figure 3 The validation of DEGs in susceptible (26M) and resistant (No. 26) cucumber at 2 dpi.
