# Supplementary figures and images for: Redox Status, JA and ET Signaling Pathway Regulating Responses to Botrytis cinerea Infection Between the Resistant Cucumber Genotype and Its Susceptible Mutant
Source: Front Plant Sci. 2020 Sep 25;11:559070. doi: 10.3389/fpls.2020.559070 (PMC7546314; doi:10.3389/fpls.2020.559070)

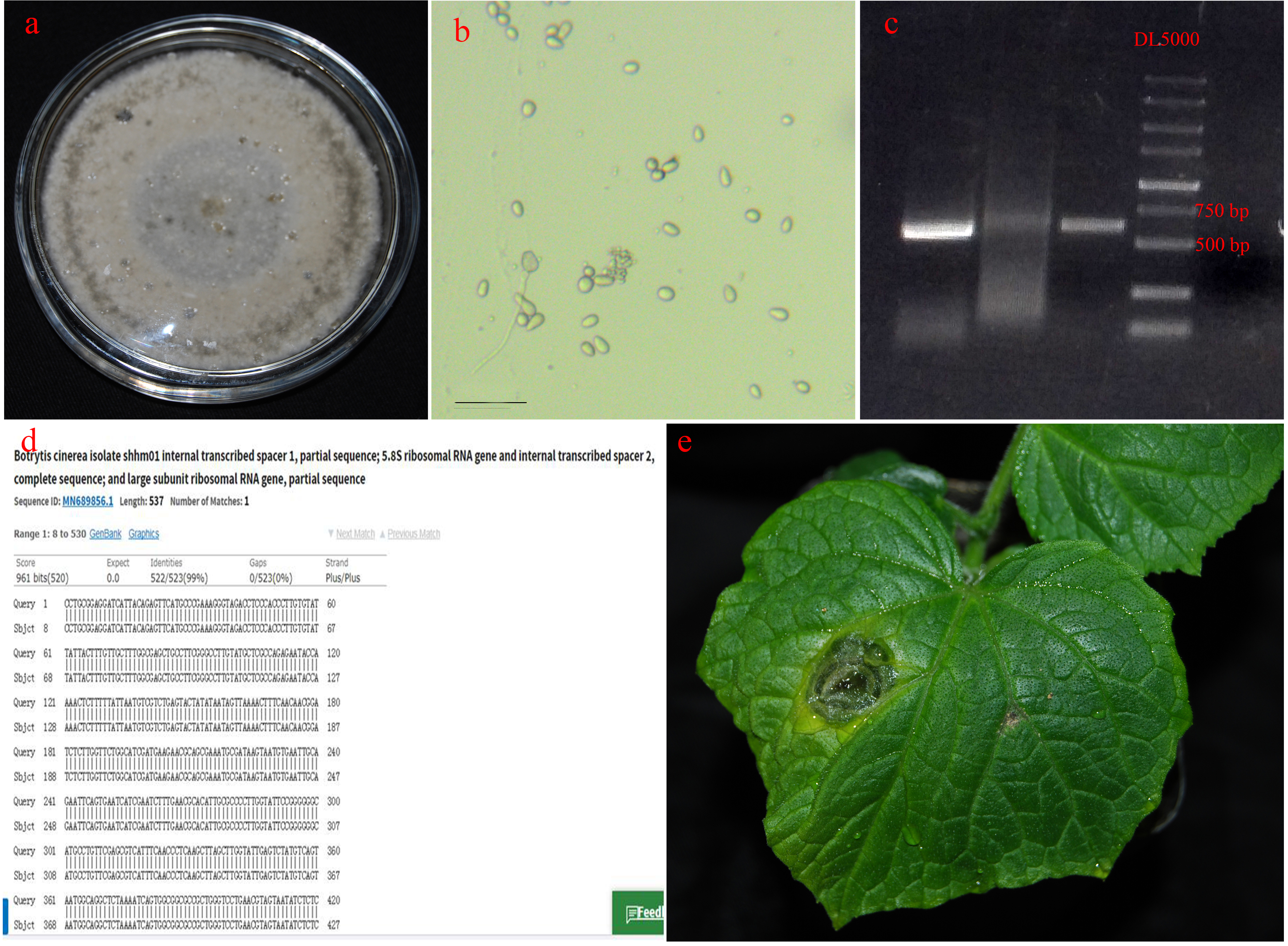

Supplement: Supplementary Figure 1 — Isolation and identification of B. cinerea. (A) the isolation of B. cinerea; (B) morphological observation of B.cinerea; (C) PCR amplification; (D) sequence alignment; (E) symptom of B. cinerea after reinfection in cucumber. [file Image_1.jpg]

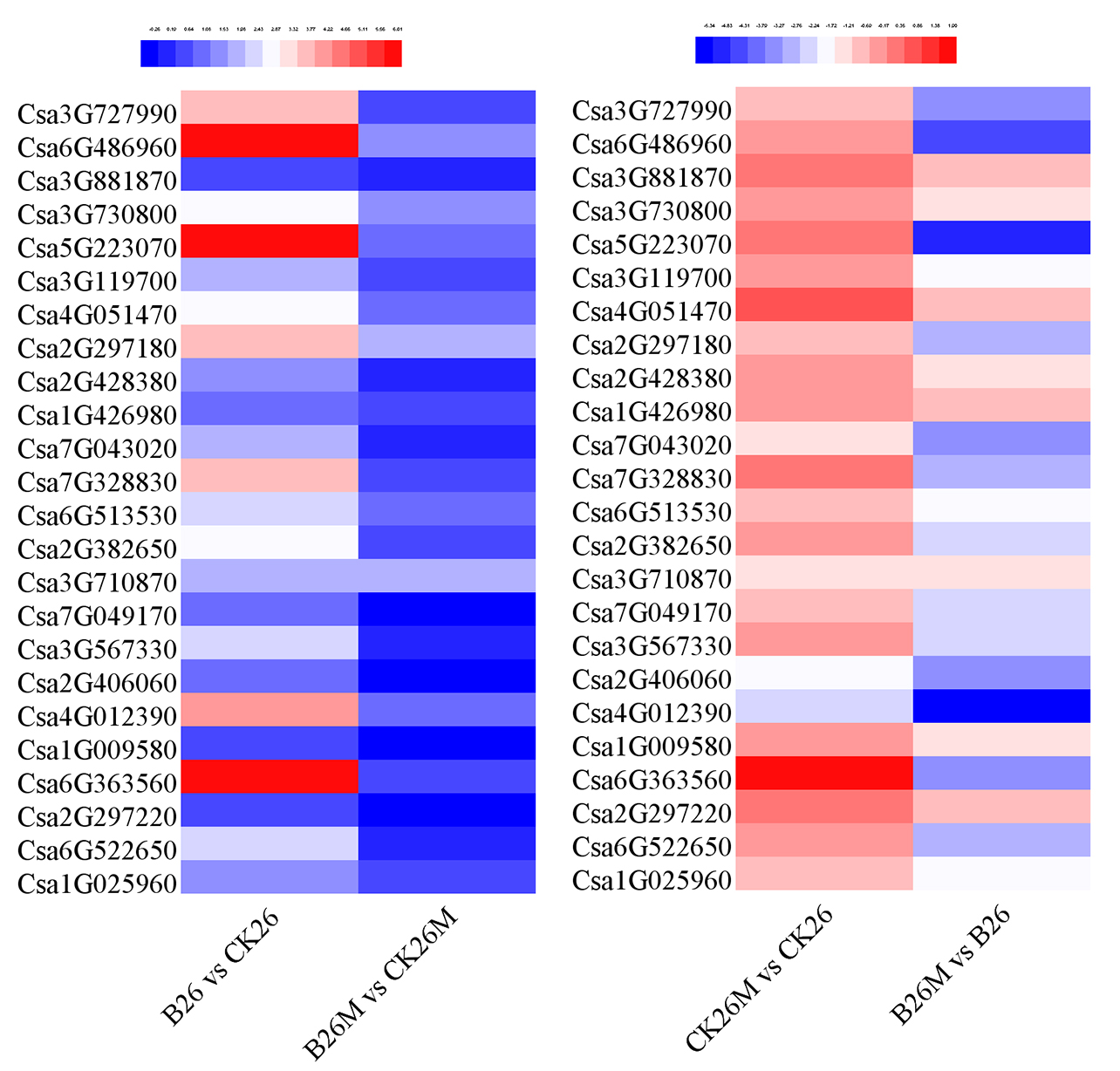

Supplement: Supplementary Figure 2 — The DEGs of WRKY in susceptible (26M) and resistant (No. 26) cucumber at 2 dpi. [file Image_2.jpg]

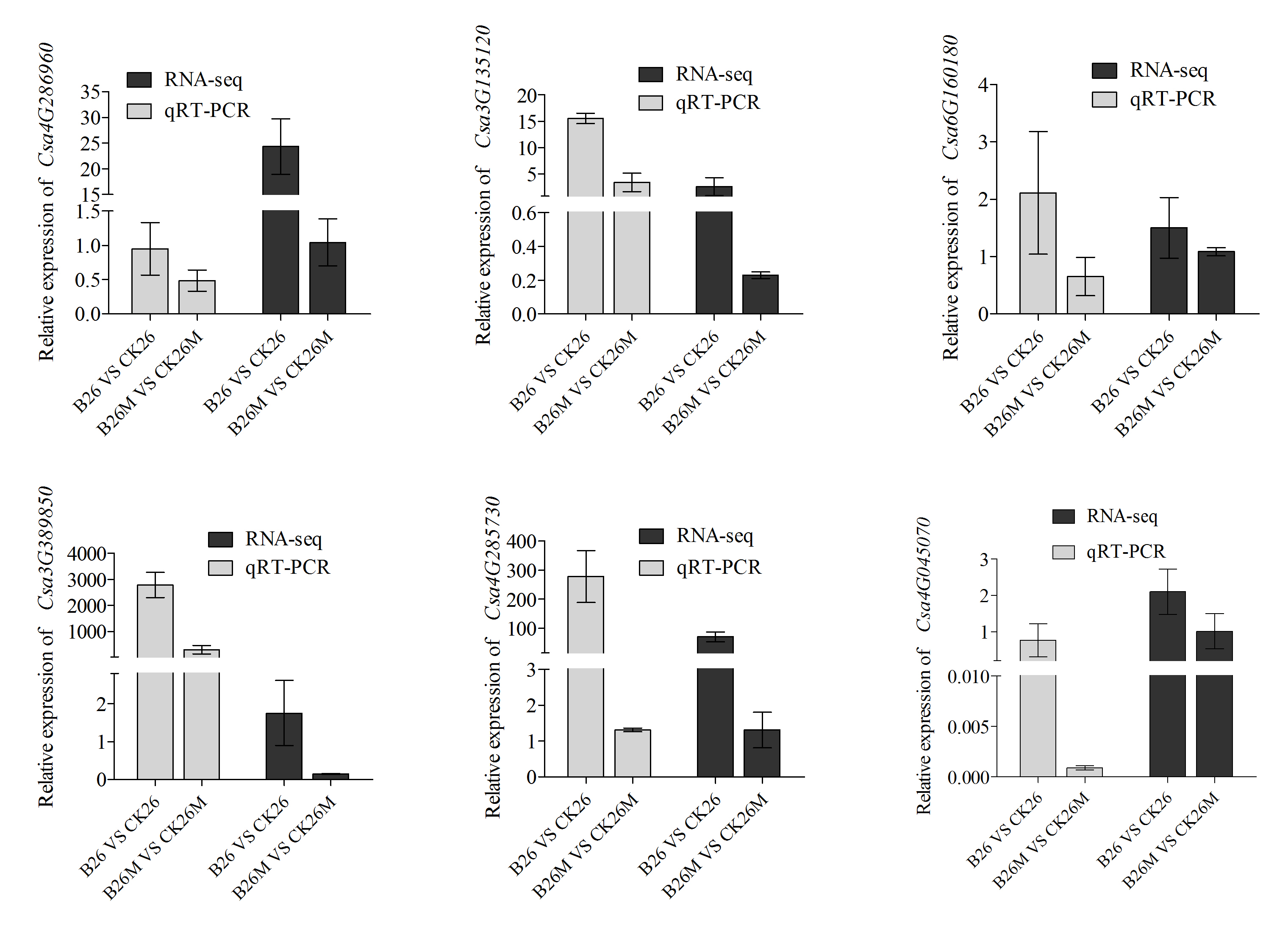

Supplement: Supplementary Figure 3 — The validation of DEGs in susceptible (26M) and resistant (No. 26) cucumber at 2 dpi. [file Image_3.jpg]
